# Molecular Targeted Therapy in the Treatment of Chordoma: A Systematic Review

**DOI:** 10.3389/fonc.2019.00030

**Published:** 2019-02-01

**Authors:** Tong Meng, Jiali Jin, Cong Jiang, Runzhi Huang, Huabin Yin, Dianwen Song, Liming Cheng

**Affiliations:** ^1^Division of Spine, Department of Orthopedics, Tongji Hospital Affiliated to Tongji University School of Medicine, Shanghai, China; ^2^Shanghai Bone Tumor Institution, Shanghai, China; ^3^Department of Orthopedics, Shanghai General Hospital, School of Medicine, Shanghai Jiaotong University, Shanghai, China; ^4^Department of Central Laboratory, Shanghai Tenth People's Hospital of Tongji University, School of Medicine, School of Life Sciences and Technology, Tongji University, Shanghai, China; ^5^Beth Israel Deaconess Medical Center, BIDMC Cancer Center, Harvard Medical School, Cancer Research Institute, Boston, MA, United States; ^6^Key Laboratory of Spine and Spinal Cord Injury Repair and Regeneration, Ministry of Education, Tongji University, Shanghai, China

**Keywords:** molecular targeted therapy, bone tumor, chordoma, systematic review, imatinib, erlotinib

## Abstract

**Objectives:** Chordoma is a rare bone malignancy that affects the spine and skull base. Treatment dilemma leads to a high rate of local relapse and distant metastases. Molecular targeted therapy (MTT) is an option for advanced chordoma, but its therapeutic efficacy and safety have not been investigated systematically. Therefore, a systematic review was conducted on studies reporting MTT regimens for chordoma.

**Methods:** Clinical trials, case series and case reports on chordoma MTT were identified using MEDLINE, Cochrane library and EMBASE, and systematically reviewed. Data on clinical outcomes, such as median overall survival, progression-free survival, response rate and adverse events (AEs) were extracted and analyzed.

**Results:** Thirty-three eligible studies were selected for the systematic review, which indicated that imatinib and erlotinib were the most frequently used molecular targeted inhibitors (MTIs) for chordoma. For PDGFR-positive and/or EGFR-positive chordoma, clinical benefits were achieved with acceptable AEs. Monotherapy is preferred as the first-line of treatment, and combined drug therapy as the second-line treatment. In addition, the brachyury vaccine has shown promising results.

**Conclusions:** The selection of MTIs for patients with advanced or relapsed chordoma should be based on gene mutation screening and immunohistochemistry (IHC). Monotherapy of TKIs is recommended as the first-line management, and combination therapy (two TKIs or TKI plus mTOR inhibitor) may be the choice for drug-resistant chordoma. Brachyury vaccine is a promising therapeutic strategy and requires more clinical trials to evaluate its safety and efficacy.

## Introduction

Chordoma is a relatively rare malignant bone tumor with an incidence of 0.08 per 100,000 (1). It accounts for 1–4% of all bone malignancies, and ~20% of primary spine tumors (2). Although it can occur at any segment of the spine, the predominant site of chordoma are fused segments like clivus and sacrococcyx (3). It is an indolent malignancy that progresses slowly, but exhibits strong local aggressiveness and often grows into huge masses that compress vital nerves and blood vessels (4). In addition, since chordoma is usually unresponsive to the conventional radiotherapy and cytotoxic chemotherapy, surgery is the primary therapeutic option (1, 5). Large case series including our previous one have shown that a total resection of the tumor, with the goal of negative microscopic margins, is crucial for long-term positive outcomes (6). However, the complex anatomy of the spine and the relatively large tumor volume make a clean resection technically challenging, leading to a high rate of local relapse and distant metastases (7). Regarding to this advanced setting, conventional therapeutic methods were shown to be not highly effective (1). Therefore, novel therapeutic strategies are needed to prolong patients' survival and improve the quality of life.

Pathologically, chordoma arises from residual notochord cells within the vertebral body (8), as verified on the basis of genetic and immuno-phenotypic biomarkers (9). New insights into the molecular mechanism underlying chordoma have also identified novel therapeutic targets (5). Molecular targeted therapy (MTT) in chordoma includes (1) imatinib and dasatinib against platelet-derived growth factor receptors (PDGFR) and stem cell factor receptor (KIT) (10, 11); (2) erlotinib, lapatinib, gefitinib, and cetuximab against epidermal growth factor receptor (EGFR) and erbB-2/human epidermal growth factor receptor 2 (HER2/neu) (12, 13); (3) sorafenib, pazopanib, and sunitinib that target angiogenic factors like vascular endothelial growth factor receptor (VEGFR) (14–16); and (4) temsirolimus and sirolimus that target the phosphoinositide 3-kinase (PI3K)/AKT/mammalian target of rapamycin (mTOR) pathway (17).

The indications for MTTs in chordoma patients are largely based on a few prospective clinical trials, small retrospective studies, and even case reports (10–17). However, the efficacy and safety of MTT regimens in chordoma patients, as well as the underlying molecular mechanisms, lack systematic investigation. Therefore, we conducted a systematic review on MTT regimens in chordoma patients to determine the clinical outcomes and underlying molecular mechanisms.

## Materials and Methods

### Search Strategy

For this systematic review, we used standard procedures from PRISMA guidelines (18). A comprehensive, systematic search was performed using MEDLINE (via PubMed), Cochrane Library and EMBASE. To find appropriate studies in MEDLINE, we used a combination of terms related to the MeSH terms “Chordoma/drug therapy” OR the free-text searching “Chordoma” AND (“targeted therapy OR inhibitor OR inhibit OR inhibition”). This search was further modified as appropriate for Cochrane Library and EMBASE. Initial search was performed on January 17, 2018 and repeated on July 1, 2018.

### Eligibility Criteria

Studies were deemed eligible for the assessment of MTTs in patients with chordoma, irrespective of previous and subsequent other treatment. Only English language publications were included. For clinical trials, case series and case reports published exclusively in abstract or news form, only those containing new data were analyzed. For literature reviews, new personal unpublished data is also included. Reference lists of selected studies and previous reviews associated with similar topics were screened manually. New clinical trials for chordoma were found from Chordoma Foundation, ClinicalTrials.gov, EU Clinical Trials Register and WHO International Clinical Trials Registry Platform. Although gray literature (such as unpublished reports, conference abstracts and dissertations) might provide some negative results and decrease the publication bias, we did not access them, because they were usually not peer reviewed and might be later published in peer-reviewed journals.

### Data Extraction and Synthesis

After removal of duplicates, titles and abstracts of all identified publications were systematically screened by two independent reviewers (MT and YHB). Discrepancies between reviewers were resolved by discussion. When eligibility criteria seemed to be met, the two reviewers (MT and YHB) independently assessed retrieved full texts and extracted information. If disagreements were still remained, the third reviewer (SDW) helped to reach an agreement. We contacted with the Chordoma Foundation in order to get helpful information. Additionally, we corresponded with researchers clarify study eligibility if the published study was unclear, although responses were poor. Extracted data were study characteristics (study design, first author, year of publication), patient characteristics (total number, history of treatment) and tumor characteristics (gene mutation and immunohistochemistry), MTT information (type of agents, dosage, course of treatment and adverse events), evaluation criteria (Choi's criteria, the response evaluation criteria in solid tumor (RECIST), clinical and radiological or metabolic response), and survival (duration of follow-up, progression-free survival and overall survival).

## Results

### Search Results

**Figure 1:**
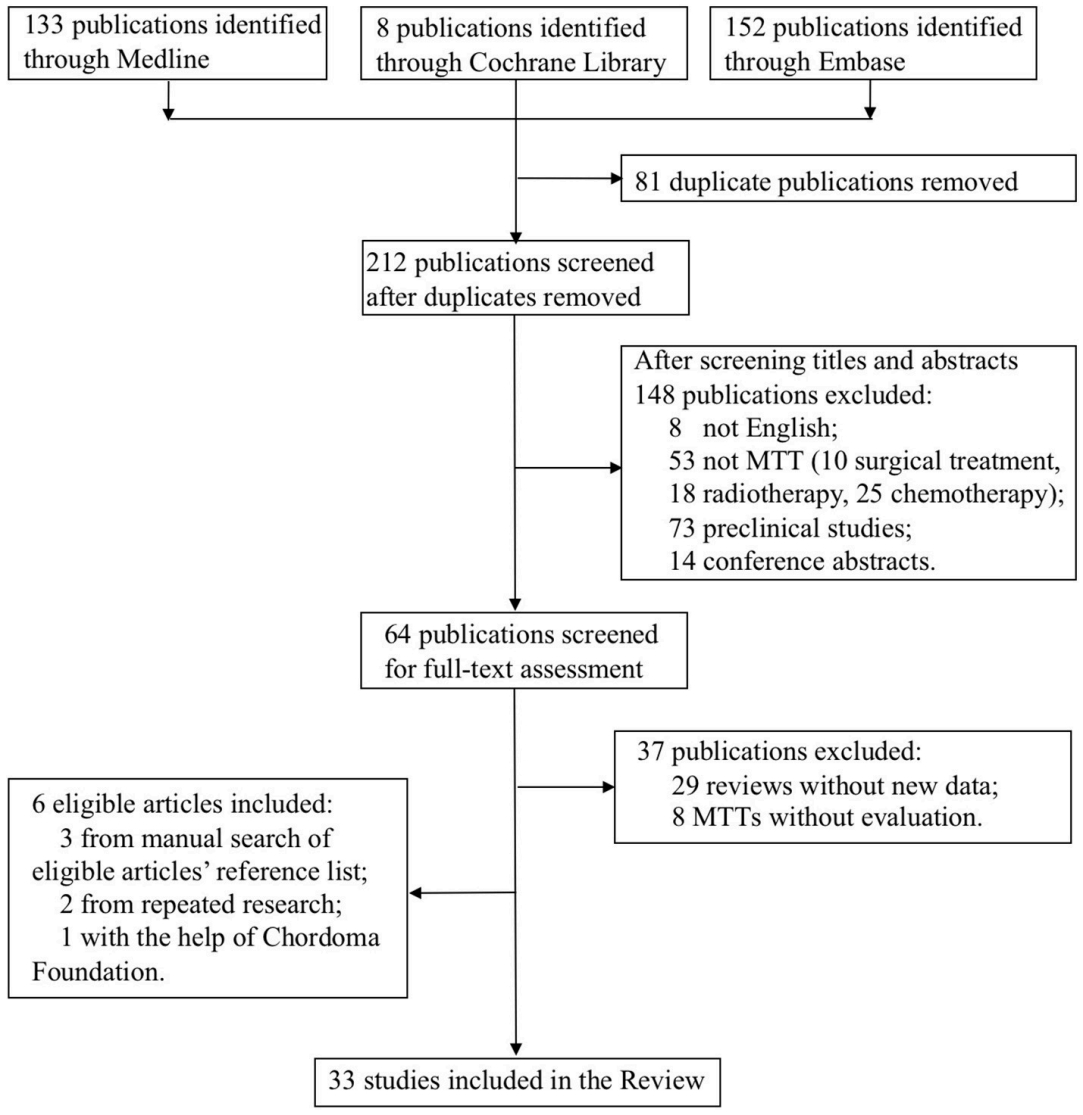
PRISMA flow diagram of the study selection process.

The flow-chart for the selection and exclusion of relevant publications is shown in Figure 1. We identified 293 studies in the initial screening, and after removing duplicates and papers based on their titles and abstracts, selected 64 publications for full-text assessment. Twenty-seven studies met our inclusion criteria, and six more were included—three from manually searching the reference list of the selected articles, two from repeated search and one with the help of the Chordoma Foundation. Finally, 33 studies were included in this systematic review.

### Study Characteristics

**Figure 2:**
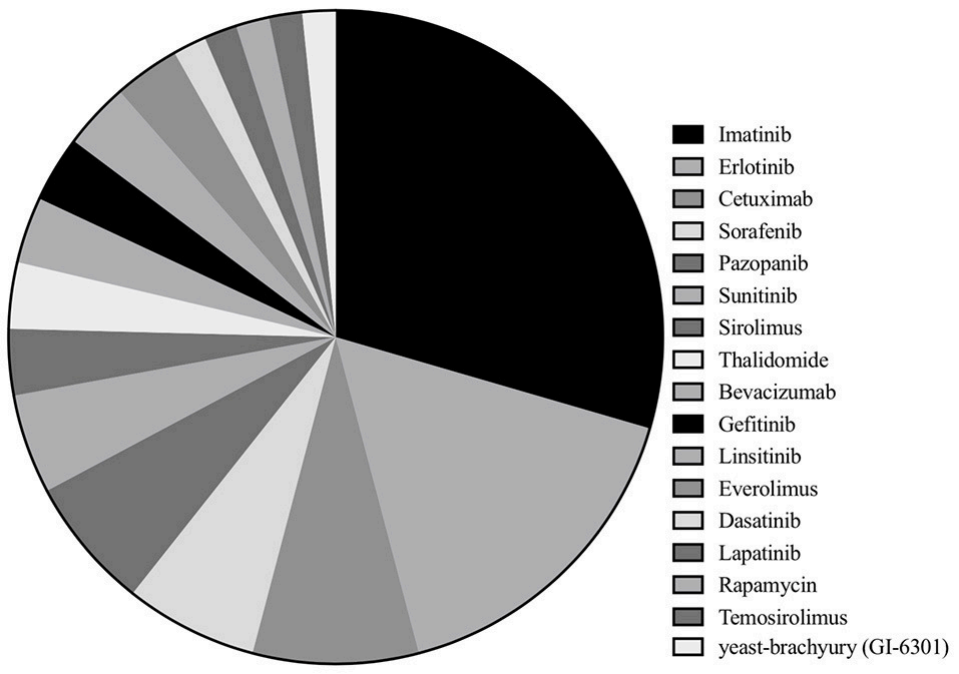
Of the included studies, the proportion of studies reporting each molecular targeted inhibitor.

**Figure 3:**
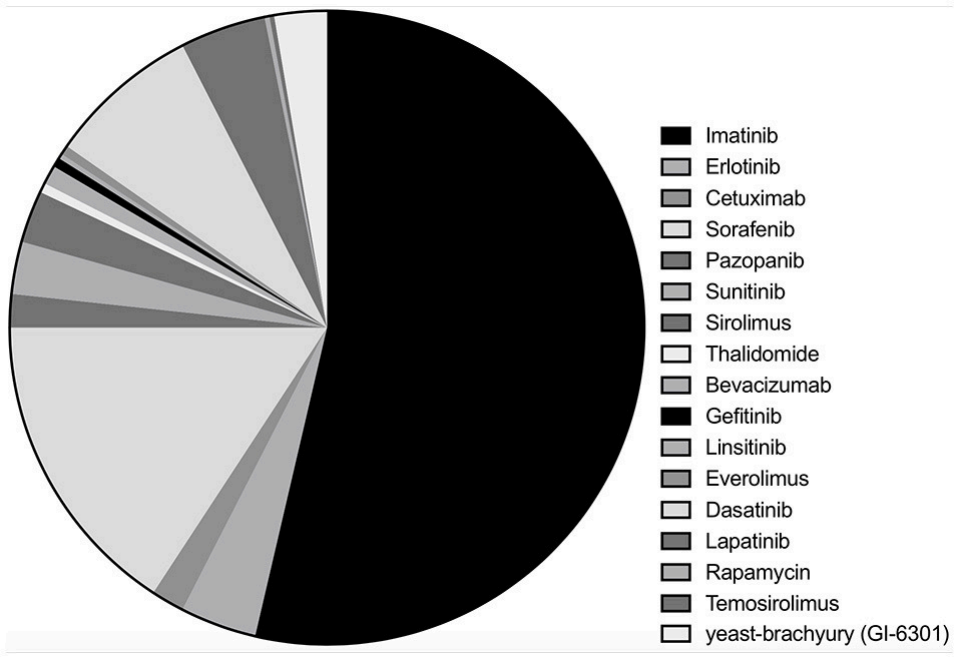
Of the included chordoma patients, the proportion of patients received each molecular targeted inhibitor.

Among 33 studies, nine studies were clinical trials (10–12, 14, 15, 19–22), with eight retrospective case series (16, 17, 23–28), and 16 case reports (13, 29–43). Imatinib was assessed in 18 studies with a total of 221 patients (10, 16, 17, 19, 23–28, 32, 34–36, 38, 39, 41, 42), erlotinib in 10 studies with 16 patients (13, 17, 22, 33, 35, 38, 40–42), cetuximab in five studies (seven patients) (13, 30, 31, 33, 41), sorafenib in four studies (65 patients) (15, 17, 21, 37), pazopanib in four studies with seven patients (16, 28, 41, 43) and sunitinib in three studies with 11 patients (14, 17, 28). Sirolimus, thalidomide, bevacizumab, gefitinib, linsitinib, and everolimus were accessed in two studies each (13, 22, 25, 28–31, 33, 34, 40–42), whereas dasatinib (32 patients) (11), lapatinib (18 patients) (12), rapamycin (one patients) (34), temosirolimus (one patients) (17) and yeast-brachyury (GI-6301) vaccine (11 patients) (20) were only analyzed in one study each (Figures 2 and 3). Monotherapy of MTTs was reported in 24 studies (10–12, 14–17, 20, 21, 23, 24, 26–28, 32, 34–39, 41, 43, 44) with combination therapy in 13 studies (13, 19, 22, 25, 28–31, 33, 39–42).

RECIST evaluation criteria was used in 19 studies (10–15, 17, 19, 20, 22, 25–28, 32, 40, 42, 43, 45) and Choi's criteria is applied in three studies (11, 12, 25). Twenty-one studies were evaluated by clinical/radiological or metabolic responses (16, 23–27, 29–43). Adverse events (AEs) were reported in 25 studies, including hematological anomalies like anemia, thrombocytopenia, as well as non-hematological AEs like fatigue, fever, anorexia, QTc prolongation, abnormal liver function, nausea, and vomiting (10–15, 19, 22, 23, 26–38, 40, 43, 45).

### Efficacy and Safety of MTT Regimens in Chordoma Patients

#### PDGFR Inhibitors (Table 1)

Imatinib mesylate (IM), a specific tyrosine kinase inhibitor (TKI) targeting PDGFR and KIT (10, 46), was the most frequently-used MTT in chordoma patients. Eighteen studies investigated the therapeutic efficacy of IM on 221 patients (10, 16, 17, 19, 23–28, 32, 34–36, 38, 39, 41, 42), including three clinical trials (10, 19, 28), seven retrospective case series (16, 17, 23–27), and eight case reports (32, 34–36, 38, 39, 41, 42). Fourteen studies (204 patients) analyzed the efficacy of imatinib as monotherapy (10, 16, 17, 23, 24, 26–28, 32, 34–36, 39, 41), of which four studies (181 patients) used RECIST and 3 were focused on PDGFRβ-expressing chordoma. In these four studies, four patients achieved partial response (PR) (2.2%), 133 cases sustained stable disease (SD) (73.5%) and 44 cases experienced progressive disease (PD) (24.3%) (10, 17, 26, 27, 32). Clinical/radiological or metabolic responses were evaluated in 13 studies (85 patients), with 33 patients achieving PR (38.8%), 23 patients sustaining SD (27.1%) and 29 patients experiencing PD (34.1%) (16, 23, 24, 26–28, 32, 34–36, 38, 39, 41). Five of the above studies (73 patients) focused on PDGFRβ-expressing chordoma, with 45.2% PR, 31.5% SD, and 23.3% PD cases (23, 24, 26, 27, 36), and eight studies included 12 patients that experienced PD within a short period of time.

**Table 1:** Molecular targeted therapy of chordomas with PDGFR inhibitors (imatinib, dasatinib).

**References**	**Study design**	**Levels of evidence**	**Sample size**	**Conditions**	**IHC positive**	**Drug (dosage)**	**Median treatment time (months)**	**AEs**	**Outcomes**			**Median follow-up (months)**	**Median PFS (months)**	**Median OS (months)**
									**Choi's criteria**	**RECIS**	**TRMR**			
Stacchiotti et al. (10)	Phase II	IV	56	Advanced chordoma	PDGFRB/ PDGFB	Imatinib (800 mg/day)	9.1	Grade 3 toxicity: 72%	—	PR: 2%; SD: 70%; PD: 28%	—	26.4	9.2	35
Casali et al. (23)	Case series	IV	6	Advanced chordoma	PDGFRB	Imatinib (800 mg/day)	—	Toxicity: 33.3%	—	—	PR: 66.6%; SD: 16.7%; PD: 16.7%	—	—	—
Hindiet al. (27)	Case series	IV	46	Advanced chordoma	PDGFB/ PDGFRB	Imatinib (800 mg/day)	—	Toxicity: 87.5%	—	PR: 0; SD: 74%; PD: 26%	PR: 40%; SD: 28%; PD: 32%	24.5	9.9	30
Geoerger et al. (24)	Case series	IV	3	Chordoma	PDGFB/ PDGFRB/KIT	Imatinib (800 mg/day)	—	—	—	—	PR: 67%; SD: 33%	—	—	—
Ferraresi et al. (26)	Case series	IV	17	Advanced chordoma	PDGFRB	Imatinib (800 mg/day)	—	—	—	SD	PR: 54%; SD + PD: 46%	—	—	—
Launay et al. (35)	Case report	V	1	Advanced chordoma	EGFR	Imatinib (600 mg/day)	—	None	—	—	PD	—	—	—
Singhal et al. (32)	Case report	V	1	Recurrent chordoma	EMA/ cytokeratins	Imatinib (600 mg/day)	—	Grade 2 skin rash	—	—	PD	—	—	—
Trapani et al. (42)	Case report	V	1	Recurrent chordoma	PDGFRB, EGFR, pS6	Imatinib (400 mg/day) + everolimus	—	—	—	PD	—	—	—	—
Stacchiotti et al. (25)	Case series	IV	10	Advanced chordoma	mTOR effectors (AKT, S6) PDGFR	Imatinib (400 mg/day) + sirolimus (2 mg/day)	9	Grade 3 toxicity 30%	PR: 78%; SD: 11%; PD: 11%	PR: 11%; SD: 78%; PD: 11%	PR: 78%	—	—	—
Lebellec et al. (17)	Case series	IV	62	Advanced chordoma	—	Imatinib	—	—	—	PR: 5%; SD: 69%; PD: 26%	—	—	—	—
Adenis et al. (19)	Phase I	IV	7	Chordoma	—	MC (50 mg two times daily) + imatinib (400 mg/day)	—	Anemia, Nausea, vomiting, Fatigue	—	Long-lasting SD, PD at last	—	—	10.2	—
Lipplaa et al. (28)	Case series	IV	2	Metastatic chordoma	VEGFR	Imatinib	9		—	—	PD	—	—	—
Lipplaa et al. (28)	Case series	IV	1	Metastatic chordoma	VEGFR	Imatinib + sirolimus	—	Grade 2 fatigue, intermittent diarrhea	—	—	PD	—	—	—
Mercier et al. (36)	Case report	V	1	Recurrent chordoma	PDGFRB	Imatinib (400–800 mg/day)	2.25	Intracranial hemorrhage	—	—	PD	—	—	—
Chay et al. (34)	Case report	V	1	Recurrent chordoma	—	Imatinib	1.25	Persistent vomiting	—	—	PD	—	—	—
Houessinon et al. (38)	Case report	V	1	Recurrent chordoma	—	Imatinib (400–800 mg/day)	5	—	—	—	PD	—	—	—
Rohatgi et al. (39)	Case report	V	2	Metastatic chordoma	—	Imatinib (800 mg/day)	—	—	—	—	PR	—	60	—
Migliorini et al. (41)	Case report	V	2	Recurrent chordoma	Brachyury, EGFR, p53	Imatinib	—	—	—	—	PD	—	—	—
Jagersberg et al. (16)	Case series	V	2	Recurrent chordoma	—	Imatinib	—	—	—	—	PD	—	9	—
Schuetze et al. (11)	Phase II	IV	32	Incurable chordoma by conventional treatments	Src family of kinases, PDGFR, KIT, ephrin	Dasatinib (50–100 mg twice daily)	4	Grade 3 toxicity: 39.7%; Grade 4 toxicity: 6.9%	OR: 18.75%	OR: 3.125%	—	—	6.3	—

*IHC, immunohistochemistry; AEs, adverse events; RMR, radiological or metabolic response; PR, partial response; SD, stable disease; PD, progressive disease; TVR, tumor volume regression; MC, metronomic cyclophosphamide*.

Progression-free survival (PFS) and overall survival (OS) are important indices of clinical outcome, and they were reported in two large case-studies (10, 27). Stacchiotti et al. conducted a phase II trial in 56 patients with chordoma, and the median PFS and OS were 9 and 35 months, respectively (10). A retrospective study on 46 chordoma patients reported a median PFS of 9.9 months (27).

AEs were reported in eight studies (10, 23, 27, 32, 34, 36), with skin rash being the most common, followed by oedema, chronic anemia, fatigue and fluid retention (10, 26). Subacute intraventricular hemorrhage was seen in one case of clivus chordoma treated with imatinib (36).

Dasatinib, an inhibitor of PDGFR and Src, was evaluated in a phase II study (NCT00464620) (11) on 32 patients. The median PFS and 6 months PFS rate were 6.3 months and 54%, respectively. The 2- and 5-years OS rate were 43 and 18%, respectively. Six patients had an objective response (OR) according to Choi criteria and one for RECIST. Fatigue, fever, anorexia, nausea, and vomiting occurred in more than 5% of the patients.

#### EGFR Inhibitors (Table 2)

Erlotinib was the most commonly used anti-EGFR agent and was analyzed in 10 studies (16 patients) for the treatment of chordoma (13, 17, 22, 32, 33, 35, 38, 40–42), including one clinical trials (22), one retrospective case study (17) and eight case reports (13, 17, 22, 32, 33, 35, 38, 40–42). Monotherapy with erlotinib was used in five studies (nine patients) (17, 32, 35, 38, 42), three (seven patients) of which were evaluated by RECIST (17, 32, 42), reporting PR in two patients and SD in five patients. Three case reports were evaluated by clinical/radiological or metabolic responses (32, 35, 38). All achieved PR and significant tumor bulk reduction was seen in two patients (70 and 46%, respectively). Skin rashes were commonly seen in the erlotinib-treated patients.

**Table 2:** Molecular targeted therapy of chordomas with EGFR inhibitors (erlotinib, linsitinib, cetuximab, lapatinib, gefitinib).

**References**	**Study design**	**Levels of evidence**	**Sample size**	**Conditions**	**IHC positive**	**Drug (dosage)**	**Median treatment time (months)**	**AEs**	**Outcomes**			**Median follow-up (months)**	**Median PFS (months)**	**Median OS (months)**
									**Choi's criteria**	**RECIST**	**RMR**			
Launay et al. (35)	Case report	V	1	Advanced chordoma	EGFR	Erlotinib (150 mg/day)	40	None	—	—	TVR (70%)	40	12	40
Singhal et al. (32)	Case report	V	1	Recurrent chordoma	EMA, cytokeratins	Erlotinib (150 mg/day)	18	Grade 2 skin rash	—	PR	TVR (46%)	18	11	18
Trapani et al. (42)	Case report	V	1	Recurrent chordoma	PDGFR-β, EGFR, pS6	Erlotinib (150 mg/day)	—	—	—	PD (4 months), SD (16 months)	—	—	—	—
Asklund et al. (13)	Case report	V	3	Recurrent chordoma	—	Erlotinib (100–150 mg/day) + cetuximab;	—	Infection	—	—	—	—	—	—
Asklund et al. (13)	Case report	V	3	Recurrent chordoma	—	Erlotinib 100–150 mg/day + bevacizumab 10 mg/kg^*^week	—	Infection	—	PR: 33%, SD: 67%	—	—	—	—
Aleksic et al. (40)	Case report	V	1	Recurrent chordoma	brachyury, EGFR, IGF-1R	Erlotinib (100 mg/day) + linsitinib (50 mg/day)	61	Toxicity ≤ grade 2	—	PR	TVR	69	60	69
Lebellec et al. (17)	Case series	IV	5	Advanced chordoma	—	Erlotinib	—	—	—	PR: 20%, SD: 80%	—	—	4	—
Macaulay et al. (22)	Phase I	V	1	Advanced chordoma	—	Erlotinib + linsitinib	—	—	—	—	—	—	—	—
Migliorini et al. (41)	Case report	V	1	Recurrent and metastatic chordoma	Brachyury, EGFR, p53	Erlotinib + cetuximab	—	—	—	—	SD	—	—	—
Asklund et al. (33)	Case report	V	1	Advanced chordoma	—	Erlotinib + cetuximab	—	—	—	—	PD	—	—	—
Asklund et al. (33)	Case report	V	1	Advanced chordoma	—	Erlotinib + bevacizumab	—	—	—	—	TVR	—	—	—
Houessinon et al. (38)	Case report	V	1	Recurrent chordoma	—	Erlotinib (150 mg/day)	28	A moderate rash and diarrhea	—	—	PR	—	28	—
Stacchiotti et al. (12)	Phase II	V	18	Advanced chordoma	EGFR and HER2/neu	Lapatinib (1,500 mg/day)	—	G ≥ 2 toxicity	PR: 33%, SD: 39%, PD: 28%	PR: 40%, SD: 50%, PD: 10%	—	10.5	6 (Choi); 8 (RECIST)	25
Lindén et al. (31)	Case report	V	1	Recurrent and metastatic chordoma	—	Cetuximab 500 mg/week + gefitinib 250 mg/day	4	Facial acne	—	—	TVR (44%)	4	4	4
Hof et al. (30)	Case report	V	1	Recurrent and metastatic chordoma	EGFR	Cetuximab 500 mg/week + gefitinib 250 mg/day	12	Facial acne, diarrhea and skin defects	—	—	TVR	12	12	12

*IHC, immunohistochemistry; AEs, adverse events; RMR, radiological or metabolic response; PR, partial response; SD, stable disease; PD, progressive disease; TVR, tumor volume regression*.

Lapatinib monotherapy was evaluated in a phase II clinical trial on 18 patients with EGFR-positive chordoma (12). Six patients achieved PR and seven sustained SD, with the median PFS of 6 months according to the Choi criteria. In contrast, all patients had SD by RECIST criteria with the median PFS of 8 months. Most patients experienced G ≥ 2 AEs.

Combined therapy with EGFR inhibitors was used in seven studies (eight patients) (13, 22, 30, 31, 33, 40, 41). Erlotinib was also the most common agents used in the combined MTT regimens (five studies, seven patients) (13, 22, 33, 40, 41).

Linsitinib, an inhibitor of IGF-1R/insulin receptor (INSR), was evaluated in a phase I study in combination with erlotinib (NCT00739453) (22). One patient with chordoma achieved PR for 18 months according to RECIST, with a PFS of 5 years. AEs included QTc prolongation, abnormal liver function, hyperglycemia and anorexia (22, 40).

The anti-EGFR monoclonal antibody (mAb) cetuximab was applied in combination with erlotinib in one patient with EGFR-positive chordoma, and he had a SD for 6 months (41). However, four patients with EGFR-negative chordoma experienced PD after receiving the same regimen. The treatment failure prompted a switch to bevacizumab, an anti-VEGF mAb (13, 33). Following this change, two patients achieved PR and another two presented SD. Treatment-related fatigue was observed in one patient (13, 33). Combined regimen of cetuximab and gefitinib was also effective in two cases of EGFR-positive chordoma (30, 31), where one achieved a PR for 9 months and the other had a 44% reduction in tumor bulk. Pronounced AEs, such as rash, acne, diarrhea, and skin defects, were reported in both cases (30, 31).

#### VEGFR Inhibitors (Table 3)

Sorafenib, a TKI against VEGFR and PDGFR, was assessed in four studies (15, 17, 21, 37). A phase II trial was conducted on 27 patients with chordomas (NCT00874874) (15), and OR was observed in one patient as per RECIST. The 12 months PFS and OS rates were 73.0 and 86.5%, respectively. In a study on 11 patients treated with sorafenib, PR was obtained in one patient, with SD in nine patients and PD in one patient according to RECIST (17). Another study assessing sorafenib reported a PFS of 12 months (37). However, sorafenib was limited by severe AEs like thrombocytopenia and diarrhea, and the rates of grade 3 and 4 toxicity were 77.8 and 14.8%, respectively.

**Table 3:** Molecular targeted therapy with VEGF and VEGFR inhibitors (sorafenib, sunitinib, thalidomide, pazopanib).

**References**	**Study design**	**Levels of evidence**	**Sample size**	**Conditions**	**IHC positive**	**Drug (dosage)**	**Median treatment time (months)**	**AEs**	**Outcomes**			**Median follow-up (months)**	**Median PFS (months)**	**Median OS (months)**
									**Choi's criteria**	**RECIS**	**TRMR**			
Bompas et al. (15)	Phase II	IV	27	Locally advanced and metastatic chordoma	—	Sorafenib (800 mg/day)	8.7	Grade 3 (77.8%); grade 4 (14.8%)	—	OR: 4%; PR: 4%; SD + PD: 92%	—	—	PFS rates: 6 m (85.3%), 9 m (73.0%), 12 m (73.0%)	OS rates: 6 m (100%), 9 m (86.5%), 12 m (86.5%)
Lebellec et al. (21)	Phase II	IV	26	Advanced chordoma	—	Sorafenib	—	—	—	—	—	—	—	—
Lebellec et al. (17)	Case series	IV	11	Advanced chordoma	—	Sorafenib	—	—	—	PR: 9%; SD: 82%; PD: 9%	—	—	—	—
Svoboda et al. (37)	Case report	IV	1	Recurrent and metastatic chordoma	—	Sorafenib (200 mg/day)	—	Thrombocytopenia; diarrhea	—	—	SD	—	12	—
George et al. (14)	Phase II	IV	9	Advanced chordoma	VEGFR, PDGFRB	Sunitinib (37.5 mg/day)	—	Grade 1 or 2 (fatigue, diarrhea, hypertension)	—	SD: 44%; PD: 56%	—	—	—	—
Lipplaa et al. (28)	Case series	IV	1	Metastatic chordoma	VEGFR	Sunitinib (37.5–50 mg/day)	—	Grade 2 nausea, fatigue	—	PR	TVR	—	27	—
Lebellec et al. (17)	Case series	IV	1	Advanced chordoma	—	Sunitinib	—	—	—	—	—	—	—	—
Schonegger et al. (29)	Case report	V	1	Recurrent and metastatic chordoma	—	Thalidomide	12	—	—	—	PD	—	—	—
Chay et al. (34)	Case report	V	1	Recurrent chordoma	—	Thalidomide (100–300 mg/day)	23+	—	—	—	TVR (more than 50%)	—	21	—
Lipplaa et al. (28)	Case series	IV	4	Unresectable or metastatic chordoma	VEGFR	Pazopanib (600–800 mg/day)	—	Grade 2 diarrhea, fatigue	—	SD: 50%; PD: 50%	—	—	8.5	—
Ribeiro et al. (43)	Case report	V	1	Recurrent chordoma	Cytokeratins, EMA and vimentin	Pazopanib (800 mg/day)	—	Grade 3 neutropenia	—	SD	TVR (23.1%)	—	15	—
Migliorini et al. (41)	Case report	V	1	Recurrent chordoma	Brachyury, EGFR, p53	Pazopanib	6	—	—	—	PD	—	—	—
Jagersberg et al. (16)	Case series	IV	1	Recurrent chordoma	—	Pazopanib	—	—	—	—	PR	—	24	—

*IHC, immunohistochemistry; AEs, adverse events; RMR, radiological or metabolic response; OR, objective response; PR, partial response; SD, stable disease; PD, progressive disease; TVR, tumor volume regression*.

Sunitinib, a multi-targeting TKI against VEGFR and PDGFR, was assessed in three studies (14, 17, 28). A phase II trial on sunitinib was conducted on nine patients (14), four of which achieved SD according to RECIST, concurrent to a qualitative decrease in tumor density, along with a median PFS of 12 months (14). Two patients treated with sunitinib had at least SD according to RECIST (17, 28), and one achieved a PR after a 27 months SD (28). The major toxicities were of grade 1 or 2 (14).

Pazopanib, another VEGFR inhibitor, was analyzed in seven patients (16, 28, 41, 43), of which four sustained SD with the median PFS of 15 months and the remaining three experienced PD. Thalidomide, an inhibitor of VEGF, was used as a second-line treatment for chordoma after failure of imatinib, rapamycin and other chemotherapy (29, 34). While one patient achieved a 50% tumor reduction, another experienced a PD (29, 34). In addition, severe toxicities of grade 3 and 4 were reported in both cases.

#### Other Molecular Targeted Inhibitors (MTIs) (Table 4)

Monotherapy with the mTOR inhibitors rapamycin and everolimus were ineffective in chordoma patients (34, 41). The combined MTT regimen of everolimus and imatinib resulted in sustained SD in one patient, with a PFS of 16 months (42). In addition, IM plus sirolimus was used in 10 patients with IM-refractory chordoma and activated mTOR (25). Nine patients were assessed, of which one achieved PR, seven sustained SD and one experienced PD according to RECIST. According to Choi criteria, seven patients achieved PR, and one sustained SD and one experienced PD. The same MTT regimen was also used against IM- and sunitinib-refractory chordoma but was not effective due to short of the mTOR expression (28). A phase I trial evaluated the effect of IM plus metronomic cyclophosphamide (MC)-based chemotherapy on 7 IM- and sunitinib-refractory chordoma patients (19). The median PFS was 10.2 months, and the 12 months PFS and OS rates were 42.9 and 85.7%, respectively according to RECIST. No dose-limiting toxicity and drug pharmacokinetic interactions were observed.

**Table 4:** Molecular targeted therapy of chordomas with other inhibitors (rapamycin, temsirolimus, yeast brachyury vaccine).

**References**	**Study design**	**Levels of evidence**	**Sample size**	**Conditions**	**IHC positive**	**Drug (dosage)**	**Median treatment time (months)**	**AEs**	**Outcomes**			**Median follow-up (months)**	**Median PFS (months)**	**Median OS (months)**
									**Choi's criteria**	**RECIS**	**TRMR**			
Chay et al. (34)	Case report	V	1	Recurrent chordoma	—	Rapamycin	2	—	—	—	PD	—	—	—
Migliorini et al. (41)	Case report	V	1	Recurrent and metastatic chordoma	Brachyury, EGFR, p53	Everolimus	—	—	—	—	PD	—	—	—
Lebellec et al. (17)	Case series	IV	1	Advanced chordoma	—	Temsirolimus	—	—	—	—	SD	—	—	—
Migliorini et al. (41)	Case report	V	1	Recurrent chordoma	Brachyury	Pembrolizumab	6	—	—	—	TVR	—	6	—
Heery et al. (20)	Phase I	IV	11	Advanced chordoma	—	Yeast-brachyury (GI-6301) vaccine (40-80 YU)	—	—	—	PR: 10%; SD: 80%; PD: 10%	—	—	8.3	—

*IHC, immunohistochemistry; AEs, adverse events; RMR, radiological or metabolic response; PR, partial response; SD, stable disease; PD, progressive disease; TVR, tumor volume regression*.

#### Brachyury Vaccine (Table 4)

A phase I dose-escalation trial using a recombinant *Saccharomyces cerevisiae* (yeast) vaccine encoding brachyury (GI-6301) was conducted on 11 patients (20), and 10 evaluable patients showed a median PFS of 8.3 months. One patient achieved PR, with eight sustaining SD and one experiencing PD at 3 months according to RECIST. Seven patients had no evidence of PD, giving a clinical benefit rate of 70% at 5 months. The most common AEs were injection site reactions.

Ongoing and planned clinical trials on chordoma MTT are listed in Table 5.

**Table 5:** Clinical trials programs of chordomas in progress.

**Official title**	**Trial registration number**	**Type**	**Medical condition:**	**Interventions**	**Mechanism**	**Sites**	**Status**
Nilotinib with radiation for high risk chordoma	NCT01407198	Phase I	Histologically confirmed chordoma	Nilotinib (daily 200–400 mg BID) Radiation therapy	A Bcr-Abl kinase inhibitor	USA	Active, not recruiting
Study of Imatinib, a platelet-derived growth factor receptor inhibitor, and LBH589, a histone deacetylase inhibitor, in the treatment of newly diagnosed and recurrent chordoma	NCT01175109	Phase I	Histologically confirmed chordoma	Imatinib + LBH589	Imatinib: a PDGFR inhibitor LBH589: a HDAC inhibitor	USA	Unknown
CDK4/6 inhibition in locally advanced/metastatic chordoma	NCT03110744 EUDRACT 2016-004660-19	Phase II	Locally advanced or metastatic chordoma refractory to tyrosine kinase inhibitors	Palbociclib	A CDK4/6 inhibitor	Germany	Recruiting
Afatinib in locally advanced and metastatic chordoma	NCT03083678	Phase II	Locally advanced or metastatic, pathologically proven, EGFR expressing chordoma	Afatinib (40 mg/day)	A Her2 and EGFR kinases inhibitor	Italy, Netherlands, UK	Not yet recruiting
Phase I safety study of stereotactic radiosurgery with concurrent and adjuvant PD-1 antibody nivolumab in subjects with recurrent or advanced chordoma	NCT02989636	Phase I	Histologically confirmed chordoma	Nivolumab Stereotactic Radiosurgery	Nivolumab: a PD-1 Antibody	USA	Recruiting
A randomized, double-blind, phase 2 trial of GI-6301 (Yeast-Brachyury Vaccine) vs. placebo in combination with standard of care definitive radiotherapy in locally advanced, unresectable, chordoma	NCT02383498	Phase II	Histologically confirmed chordoma	GI-6301 Vaccine (Yeast-Brachyury) GI-6301 Placebo Radiotherapy	A heat-killed, recombinant yeast-based vaccine engineered to express the transcription factor, Brachyury	USA	Recruiting
Phase II trial of the immune checkpoint inhibitor nivolumab in patients with select rare CNS cancers	NCT03173950	Phase II	Primary brain sarcoma including chordoma	Nivolumab	A PD-1 Antibody	USA	Recruiting
A phase II trial of dasatinib in advanced sarcomas	NCT00464620	Phase II	Unresectable, recurrent, or metastatic soft tissue or bone sarcoma including chordoma	Dasatinib (70 mg, twice daily)	An inhibitor of Src family of kinases, PDGFR, KIT, ephrin	USA	Active, not recruiting
DART: dual anti-CTLA-4 and anti-PD-1 blockade in rare tumors	NCT02834013	Phase II	Rare tumors including chordoma	Ipilimumab Nivolumab	Ipilimumab: a CTLA-4 inhibitor Nivolumab: a PD-1 inhibitor	USA	Recruiting
A phase II, multicenter study of the EZH2 inhibitor tazemetostat in adult subjects with INI1-negative tumors or relapsed/refractory synovial sarcoma	NCT02601950 EUDRACT 2015-002469-41	Phase II	Poorly differentiated chordoma (or other chordoma with sponsor approval)	Tazemetostat (800 mg BID)	An EZH2 Inhibitor	USA, Australia, Belgium, Canada, France, Germany, Italy, Taiwan, UK	Recruiting
A phase 1 study of the EZH2 inhibitor tazemetostat in pediatric subjects with relapsed or refractory INI1-negative tumors or synovial sarcoma	NCT02601937 EUDRACT 2015-002468-18	Phase I	INI1-negative tumors including chordoma	Tazemetostat	An EZH2 Inhibitor	USA, Australia, Canada, Denmark, France, Germany, Italy, Netherlands, UK	Active, not recruiting
A phase II multi-arm study to test the efficacy of immunotherapeutic agents in multiple sarcoma subtypes	NCT02815995	Phase II	Advanced and/or metastatic sarcoma including chordoma	Durvalumab Tremelimumab	Durvalumab: a PD-L1 inhibitor; Tremelimumab: a CTLA-4 inhibitor	USA	Recruiting
A randomized phase II study of Durvalumab (MEDI4736) and Tremelimumab compared to doxorubicin in patients with advanced or metastatic soft tissue sarcoma.	EUDRACT 2016-004750-15	Phase II	Advanced or metastatic soft tissue sarcoma including chordoma	Durvalumab Tremelimumab	Durvalumab: a PD-L1 inhibitor; Tremelimumab: a CTLA-4 inhibitor	Germany	Ongoing
A phase I, open-label, multiple-ascending dose trial to investigate the safety, tolerability, pharmacokinetics, biological and clinical activity of MSB0011359C in subjects with metastatic or locally advanced solid tumors and expansion to selected indications	NCT02517398	Phase I	Solid tumors including chordoma	MSB0011359C (M7824)	A PD-L1 inhibitor	USA	Recruiting
An open-label phase 1 trial to evaluate the safety and tolerability of a Modified Vaccinia Ankara (MVA) priming followed by fowlpox booster vaccines modified to express brachyury and T-cell costimulatory molecules (MVA-BN-Brachyury/FPV-Brachyury)	NCT03349983	Phase I	Metastatic or unresectable locally advanced malignant solid tumors including chordoma	MVA-BN-Brachyury FPV-Brachyury	A brachyury vaccine	USA	Recruiting
An open phase I clinical study assessing safety and tolerability of MVX-ONCO-1 in patients with solid tumor who are not/not any longer amenable to standard therapy	NCT02193503	Phase I	—	MVX-ONCO-1	An autologous tumor vaccine	Switzerland	Recruiting
Secured access to pembrolizumab for adult patients with selected rare cancer types	NCT03012620 EUDRACT 2016-002260-14	Phase II	Unresectable, recurrent, or metastatic soft tissue or bone sarcoma including chordoma	Pembrolizumab	A PD-L1 inhibitor	France	Recruiting
A randomized phase II, placebo-controlled, multicenter study evaluating efficacy and safety of regorafenib in patients with metastatic bone sarcomas	NCT02389244	Phase II	Advanced metastatic cancer in progression including chordoma	Regorafenib Placebo	A multi-kinase inhibitor; angiogenesis inhibitor	France	Recruiting
Phase 2 study on imatinib in combination with RAD001 in advanced chordoma	EUDRACT 2010-021755-34	Phase II	PDGFRB and mTOR (or S6 or 4BP1) positive advanced chordoma	Imatinib Everolimus	Imatinib: a PDGFRB inhibitor Everolimus: a mTOR inhibitor	Italy	Ongoing
A phase 2, single arm, European multi-center trial evaluating the efficacy of afatinib as first-line or later-line treatment in advanced chordoma.	EUDRACT 2016-002766-31	Phase II	Metastatic or unresectable chordoma	Afatinib	A Her2 and EGFR kinases inhibitor	Netherlands	Ongoing
Phase II study of lapatinib in EGFR/HER2NEU positive advanced chordoma	EUDRACT 2009-014456-29	Phase II	Advanced EGFR/Her2Neu positive chordoma	Lapatinib	EGFR/Her2Neu inhibitor	Germany	Ongoing

*PDGFR, platelet-derived growth factor receptor; HDAC, histone deacetylase; Her2, human epidermal growth factor receptor 2; EGFR, epidermal growth factor receptor; PD-1, programmed cell death protein 1; CTLA4, cytotoxic T lymphocyte antigen 4*.

## Discussion

Novel therapeutic strategies against chordoma are urgently needed to prolong the overall survival and relieve symptoms. Elucidation of the underlying molecular mechanisms of chordoma have helped identify numerous potential therapeutic targets (47, 48), and several anti-chordoma agents are currently being tested in animal models and clinical trials. This systematic review is focused on the pharmacological management of chordoma patients and the clinical outcomes. Furthermore, the molecular mechanisms of MTT action have also been assessed.

### Molecular Targets

Chordoma is a genetically heterogeneous tumor with frequent imbalances of large chromosomal regions. Somatic duplications of the notochordal transcription factor brachyury (47, 48), chromosomal copy loss of phosphatase and tensin homolog (PTEN) (49), tuberous sclerosis complex (TSC) (50), cyclin-dependent kinase inhibitor 2A and 2B (CDKN2A and CDKN2B) (51), SMARCB1 (49), and PIK3CA (9) mutations are key aspects of chordoma pathogenesis, and therefore potential targets.

RTKs are the key players in the development and progression of chordoma, and their mutated forms can activate signaling cascades resulting in dysregulation of many essential proteins. Therefore, mutational analyses and IHC can greatly assist oncologists to determine the optimal inhibitors (52–56). It needs to be emphasized that mutations in the molecular targets are clinically more relevant than their immunoreactivity, since target overexpression is not always driven by the activation of the corresponding signaling pathway. For example, high levels of EGFR in the chordoma cell line JHC7 was not accompanied by activated EGFR signaling (57).

### Indications and Evaluation Criteria for MTTs

MTTs are not the first treatment options for chordoma, and only recommended for advanced or recurrent chordoma that are unresponsive to either surgical resection or radiotherapy.

The outcomes of MTTs is often difficult to evaluate in chordoma. Choi's criteria is based on changes in tumor size and density following contrast administration in CT or MRI (58). A radiological PR is defined as ≥10% decrease in tumor size or ≥15% decrease in tumor density/contrast enhancement in CT/MRI. RECIST defines PR as ≥20% decrease in tumor growth, which occurs later than that required for Choi criteria. Therefore, RECIST is not fully adequate to evaluate the clinical response in chordoma (59). Clinical/radiological and metabolic responses include symptom relief, anti-tumor effects (such as liquefaction) and changes in tumor density in the CT scan, reduction in contrast enhancement in MR, and maximum standardized uptake (SUVmax) in PET (23). However, typical tumor tissue characteristics like component and scirrhosity may also affect tumor-related symptoms, even in the absence of any changes in tumor size, resulting in incorrect readings.

### MTTs for Chordoma

Imatinib was the first effective agent tested against chordoma, and is currently the most commonly used MTIs (23). Most patients with PDGFRβ-positive chordoma benefited from imatinib treatment and avoided rapid PD, likely due to tumor necrosis and intra-tumoral subacute bleeding that manifest as liquefaction (36). A dosage of 800 mg/day is recommended, except in cases of high toxicity. The major AEs associated with imatinib include oedema, chronic anemia, fatigue and even subacute intraventricular hemorrhage (36).

Several trials have also reported the ineffectiveness of imatinib in chordoma (19, 28, 32, 35, 38, 42). In such cases, EGFR inhibitor is the second line of treatment, since PDGFRβ activation can also stimulate EGFR, given an EGFR gene copy number gain (CNG) or strong intra-tumoral EGFR staining is detected. Around 40% of chordoma patients show CNG of the chromosome band 7p12, where EGFR is located. Erlotinib has shown a good clinical effect EGFR-positive chordoma, and could serve as the second choice for imatinib-refractory chordoma (32, 35). The combination of gefitinib and cetuximab, two other inhibitors of EGFR, showed improved clinical benefits and decreased AEs (30, 31).

HER2/neu is involved in EGFR dimer formation, and the possibility of heterodimerization increases the sensitivity of EGFR-positive chordoma to 54% (60). Lapatinib, a bi-specific inhibitor blocking both EGFR and HER2/neu, achieved 33.3% PR and 38.9% SD as per Choi criteria and 100% SD according to RECIST in EGFR-positive chordoma (12). Afatinib, another bi-specific inhibitor of EGFR and HER2/neu, was the only agent which showed cytotoxic effects across multiple chordoma cell lines in a drug sensitivity assessment (57). On this basis, a new clinical trial on the effects of afatinib is currently enrolling patients (NCT03083678).

IGF signaling is also important in chordoma tumorigenesis, since IGF-1 and IGF-1R have been detected in 92 and 76% of chordoma tissues (61), and are absent in benign notochordal cell tumor and fetal notochord (52). Linsitinib, an IGF-1R inhibitor, was assessed in two studies (22, 40), and effectively controlled chordoma progression in combination with erlotinib (22, 40).

VEGF levels are significantly higher in chordoma tissues and associated with angiogenesis (62). Five VEGFR or VEGF inhibitors (sorafenib, sunitinib, pazopanib, thalidomide, bevacizumab) were evaluated in this systematic review. Although occasional severe AEs were observed occasionally, sorafenib, sunitinib, and pazopanib monotherapy resulted in substantial clinical effects. Although thalidomide was effective against drug-resistant chordomas, severe toxicities limit its clinical application. Bevacizumab can be used as a supplement for erlotinib in drug-resistant chordomas, and their combination showed good clinical effect and high tolerance. A new phase II trial evaluating the efficacy and safety of regorafenib, a multi-kinase inhibitor of VEGFR, is ongoing in France for metastatic bone sarcoma (NCT02389244).

Chordomas with indication of anti-RTK agents may also relapse or progress early. In TKI-resistant chordomas, p-AKT is a relative reliable indicator and its persistent expression following tyrphostin treatment resulted in relapse and progression (54). AKT is activated by mTOR, its downstream molecules (RPS6 and eIF4E), and Stat3. The combination of the antagonists of upstream RTKs and downstream mTOR/PI3K/MAPK/Stat not only synergistically reduced chordoma growth by avoiding the negative feedback loop (63) and PI3K-dependent feedback loop (64), but also significantly decreased the cytotoxicity of either agent (65). For example, monotherapy of rapamycin or everolimus was ineffective against tumor progression (34, 41), while combining imatinib with everolimus or sirolimus induced good clinical effects in 3 studies (12 patients) (25, 28, 42). Therefore, the combined therapy can be considered for drug-resistant chordoma.

Mutations in the downstream effectors of RTKs, like PTEN and PIK3CA, also impair TKI response (66, 67). PTEN deficient chordoma cell lines exhibit increased proliferation, reduced apoptosis and enhanced migration in chordoma cell lines (68). Reintroduction of PTEN in tumor cells increased their therapeutic sensitivity to PDGFR inhibitors, and the combination of histone deacetylase (HDAC) and PDGFR inhibitors effectively reduced the growth and invasion of chordoma cells, irrespective of PTEN status (69). On this basis, a new phase I trial of Imatinib and LBH589 (a HDAC inhibitor) is ongoing in chordoma patients (NCT01175109).

Chordomas frequently show deletions in the SMARCB1 locus (49). SMARCB1 directly antagonizes the histone methyltransferase EZH2 and regulates the cell-cycle by activating CDKN2A (45). A phase I trial on the EZH2 inhibitor tazemetostat, confirmed complete or partial responses were observed in two children with chordoma according to RECIST (NCT02601937) (45). Therefore, another phase II clinical trial on tazemetostat is ongoing in patients with SMARCB1/INI1 deleted chordoma (NCT02601950).

The loss of chromosome 9 or 9p region, which contains CDKN2A, has been reported in some chordoma patients (51). The inactivation of CDKN2A universally activates the CDK4/6 and Rb pathways (70), which are highly expressed in the chordoma tissues (71). The CDK4/6 inhibitors palbociclib and LY2835219 inhibited chordoma cell growth and proliferation *in vitro* efficiently (72, 73). A phase II clinical trial on palbociclib is currently enrolling patients with chordoma (NCT03110744).

Somatic duplications of the notochordal transcription factor brachyury was demonstrated in chordoma, and enhanced tumor growth by activating YAP (9, 47, 48). Preclinical studies have shown that a recombinant *Saccharomyces cerevisiae* (yeast) vaccine encoding brachyury (GI-6301) activates human T cells *in vitro*. A phase II GI-6301 dose-escalation trial showed a 70% clinical benefit rate in chordoma patients (20). A phase II clinical trial on the combination of GI-6301 and radiotherapy is currently enrolling chordoma patients in the United States (NCT02383498). Additionally, a phase I trial of a Modified Vaccinia Ankara (MVA)-brachyury and a fowlpox (FPV)-brachyury vaccines is currently ongoing in patients with solid tumors, including chordoma (NCT03349983).

### Limitations

In order to decrease the selection bias, this systematic review screened all published studies enrolling chordoma patients treated with MTT, including clinical trials, case series and even case reports, and provides the most detailed information. However, there were some limitations that need to be addressed. We included case reports on account of the rarity of chordoma and the paucity of available studies. However, a case report might overemphasize the final results due to lack of strong results. In addition, we only included English language publications which can also increase the selection bias. Furthermore, the baseline conditions of the patients and the evaluation criteria were not consistent across studies which is another factor contributing to selection bias. Therefore, large prospective randomized clinical trials are warranted to help clinicians determine the optimum treatment modality for chordoma patients.

## Conclusions

The selection of MTIs for patients with advanced or relapsed chordoma should be based on gene mutation screening and immunohistochemistry (IHC). Monotherapy of TKIs is recommended as the first-line treatment. Combined therapy (two TKIs or TKI plus mTOR inhibitor) may be the choice for drug-resistant chordoma. Brachyury vaccine is a promising therapeutic strategy and requires more clinical trials to evaluate its safety and efficacy.

## Author Contributions

TM did the literature search, data and data analysis, and led the writing of the review. HY, JJ, and RH contributed to the design, data collection, and analysis. HY, DS, and LC contributed their experience of clinical practice in chordoma to ensure the relevance of findings. JJ and CJ contributed their experience in the discussion of the molecular mechanism underlying chordoma and drug interaction.

### Conflict of Interest Statement

The authors declare that the research was conducted in the absence of any commercial or financial relationships that could be construed as a potential conflict of interest.
